# The optimization of the vault-predicting formula based on the anterior segment measurements from artemis insight 100

**DOI:** 10.1038/s41598-024-64390-0

**Published:** 2024-06-10

**Authors:** Hao Wang, Bo Zhang, Wenwen Du, Zaohe Sun, Qi Fan, Chenjiu Pang

**Affiliations:** 1 Department of Refractive Surgery, Henan Provincial People’s Hospital, No. 7 Weiwu Road, Zhengzhou, 450003 China; 2 Department of Refractive Surgery, Henan Eye Hospital, No. 7 Weiwu Road, Zhengzhou, 450003 China; 3 Department of Refractive Surgery, People’s Hospital Affiliated to Zhengzhou University, No. 7 Weiwu Road, Zhengzhou, 450003 China; 4 Department of Refractive Surgery, People’s Hospital Affiliated to Henan University, No. 7 Weiwu Road, Zhengzhou, 450003 China

**Keywords:** Refractive errors, Vision disorders

## Abstract

To optimize and evaluate the accuracy of the vault-predicting formula generated from a very high-frequency digital ultrasound robotic scanner (Artemis Insight 100). The relationship between the achieved lens vault (LVa) at one month after intraocular collamer lens (ICL) implantation surgery and the predicted vault (LVp) was analyzed by a retrospective study, and an optimized formula was built up. Then, the accuracy of the optimized vault-predicting formula was evaluated in a prospective study by comparing the LVa and the predicted vault from the optimized formula (LVop). The retrospective study included 77 patients (133 eyes) while the prospective study enrolled 90 patients (170 eyes). The difference between LVp and LVa at one month after surgery was statistically significant (*P* < 0.05), and the linear regression analysis of LVa against LVp yielded a good fit (*R*^*2*^ = 0.68). The optimized vault-predicting formula was LVop (μm) = 1.21 × LVp (μm) + 124.73. In the validation study, the difference between LVop and LVa was not statistically significant (*P* = 0.10), and a good agreement between LVop and LVa was shown by Bland–Altman analysis. The optimized vault-predicting formula could predict the actual LV after ICL implantation surgery, help to select an appropriate ICL size and reduce the need for re-operation.

## Introduction

The implantable collamer lens (ICL) (STAAR Surgical AG, Nidau, Switzerland) is a widely used sulcus-supported intraocular lens (IOL) designed to correct refractive errors. While the ICL was initially recommended for the surgical correction of high-degree myopia^1^, its application was expanded to correct astigmatism, low to moderate myopia, and hyperopia over the years^2,3^. The new type of ICL with a small central hole (V4c, KS-AquaPORT, STAAR Surgical AG, Switzerland) was developed by Shimizu et al. recently^4^. The V4c ICL has been proved to be safe and effective with a low rate of complications, especially minimizing the risk of acute angle-closure glaucoma in the early postoperative stage by facilitating the flow of aqueous humor over the crystal lens through the central hole^5–7^.

Despite the considerably reduced number of reported complications, the distance between the ICL and the anterior crystalline lens surface (hereafter referred to as the vault) remains a critical parameter in success of surgery evaluation. The most important factor determining the vault is the lens size of the ICL. Therefore, appropriate sizing of the ICL and precise prediction of the vault are the cornerstone for the long-term safety and stability of ICL in the eye^8,9^. An oversized ICL can irritate the iris, cause pupillary block, and lead to angle-closure glaucoma, whereas an undersized ICL may contribute to intraocular lens rotation, anterior lens opacification and induce early cataract^10,11^. Very high or very low vault may necessitate an ICL exchange^12^. Despite the best efforts to order a perfect-fit lens, almost 2.6% of implanted ICLs have improper vaulting and require exchange^13,14^.

Sizing techniques for the ICL have developed over time with the advent of new diagnostic technology and formulas. Previous methods to determine the vaulting include white-to-white (WTW, manually or with imaging systems) distance or sulcus-to-sulcus (STS) diameter using high-frequency ultrasound B-scan biometry (UBM)^15,16^. An online calculation and ordering system (OCOS) was provided by the manufacturer (STAAR Surgical AG, Nidau, Switzerland), which uses the WTW diameter, and the anterior chamber depth (ACD) for recommending the ICL size. Unfortunately, this methodology leads to about 20% of cases ending up outside the accepted vault range (< 250 μm or > 1000 μm)^14,17^. Because the four footplates of the ICL need to be placed in the ciliary sulcus, the accurate measurement of STS diameter has an important effect on the choice of the ICL size^18^. A formula for calculation of the ICL size using STS measurements has also been proposed^19,20^. However, a meta-analysis by Packer^21^ concluded that there is no definitive calculation method for ICL sizing.

The aim of the current study was to optimize the formula for predicting the ICL vault from Reinstein et al.^22^ using a very high-frequency (VHF) digital ultrasound robotic scanner. The results might yield a better prediction of ICL vaulting and avoid the need for early ICL exchange.

## Results

### The characteristics of patients

A total of 77 patients (133 eyes) were enrolled in the vault-predicting formula optimization study, while 90 patients (170 eyes) participated in the validation study of the optimized vault-predicting formula. Patient demographics, ICL characteristics, and biometric parameters of the anterior segment for the two groups are presented in Table 1. Unfortunately, no cases of 13.7 mm were included in this study because the eye size of Asian patients is generally small^23^.

**Table 1:** Descriptive statistics of the patients for optimization and validation studies of the vault-predicting formula.

Characteristics	Optimization study	Validation study
Number of patients (eyes) Sex (male), n (%)	77 (133) 32 (41.6%)	90 (170) 39 (43.3%)
Age (years), median (range)	26.58 (20, 39)	27.21 (20, 41)
Preoperative diopter SE (D), median (range)	− 8.81 (− 5.0, − 18.0)	− 9.07 (− 4.75, − 18.0)
Preoperative CDVA (LogMar), median (range)	0 (0, 0.5)	0 (0, 0.5)
Preoperative IOP (mmHg), median (range)	17 (11, 21)	16 (13, 22)
Preoperative axial length (mm), mean ± SD	26.82 ± 1.46	27.01 ± 1.29
Preoperative corneal ECD (cells/mm^2^), mean ± SD	2813.51 ± 277.62	2841.73 ± 265.49
Preoperative WTW (mm), median (range)	11.52 (10.8, 12.6)	11.61 (10.6, 12.7)
Preoperative SPD (mm), median (range)	6.39 (4.6, 7.8)	6.43 (4.7, 8.0)
Preoperative measurements by Artemis Insight100		
ACD (mm), median (range)	3.24 (2.80, 3.74)	3.29 (2.80, 3.65)
ACA (degree), median (range)	36.55 (20.41, 57.92)	38.72 (21.53, 55.49)
ACW (mm), median (range)	11.43 (10.46, 12.27)	11.49 (10.42, 12.41)
ATA (mm), median (range)	11.56 (10.62, 12.49)	11.63 (10.51, 12.74)
CLR (mm), median (range)	0.09 (− 0.39, 0.51)	0.03 (− 0.43, 0.48)
STS (mm), median (range)	11.88 (10.92, 12.83)	11.79 (10.74, 13.10)
STSL (mm), median (range)	0.47 (− 0.07, 0.69)	0.51 (− 0.11, 0.94)
CBID (mm), median (range)	10.92 (9.49, 11.79)	10.83 (9.27, 11.94)
ICL degree SE (D), median (range)	− 10.0 (− 6.0, − 18.0)	− 10.5 (− 5.5, − 18.0)
Lens vault at 1 month after surgery (μm), mean ± SD	566.44 ± 147.55	579.32 ± 137.85
Visual acuity at 1 month after surgery (LogMar), median (range)	− 0.1 (− 0.2, 0.2)	− 0.1 (− 0.2, 0.4)
Diopter SE at 1 month after surgery (D), median (range)	0.5 (− 1.0, 1.25)	0.25 (− 1.5, 1.0)
IOP (mmHg) at 1 month after surgery, median (range)	18 (10, 22)	16 (12, 22)

*SE* spherical equivalent, *CDVA* corrected distance visual acuity, *IOP* intraocular pressure, *ECD* endothelial cell density, *WTW* white-to-white distance, *SPD* scotopic pupil diameter, *ACD* anterior chamber depth, *ACA* anterior chamber angle, *ACW* anterior chamber width, *ATA* angle-to-angle diameter, *CLR* crystal lens rise, *STS* sulcus-to-sulcus diameter, *STSL* sulcus-to-sulcus lens rise, *CBID* ciliary body inner diameter.

### The optimization of the vault-predicting formula

**Figure 1:**
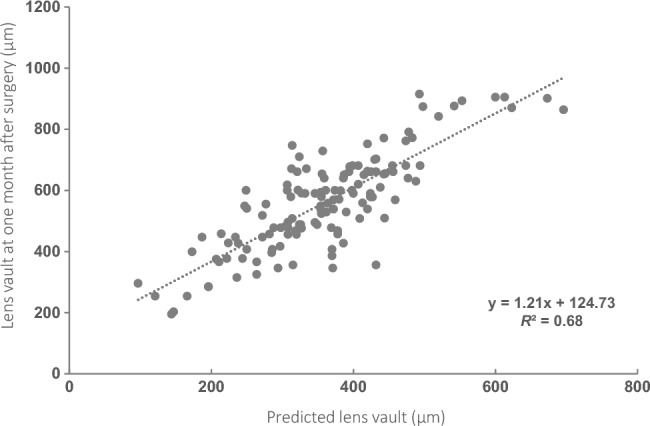
Linear correlation between predicted lens vault and the lens vault at one month after surgery. X-axis: predicted lens vault; Y-axis: lens vault at one month after surgery.

In the optimization study, the mean predicted LV (LVp) was 355.25 ± 101.58 μm, and the mean achieved post-operative LV (LVa) at one month after the surgery was 566.44 ± 147.55 μm, which were statistically significant (*P* < 0.01). Furthermore, linear regression analysis of LVa at one month against LVp yielded a very good fit with an *R*^*2*^ = 0.68, a slope of 1.21, and an intercept of 124.73 (*P* < 0.0001) (Fig. 1). The optimized predicted vault is abbreviated as LVop, yielding the following optimized vault-predicting formula:

$${\text{LVop }}\left( {{\mu m}} \right)\, = \,1.21\, \times \,{\text{LVp }}\left( {{\mu m}} \right)\, + \,124.73.$$

### The Validation of the Optimized Vault-Predicting Formula

**Figure 2:**
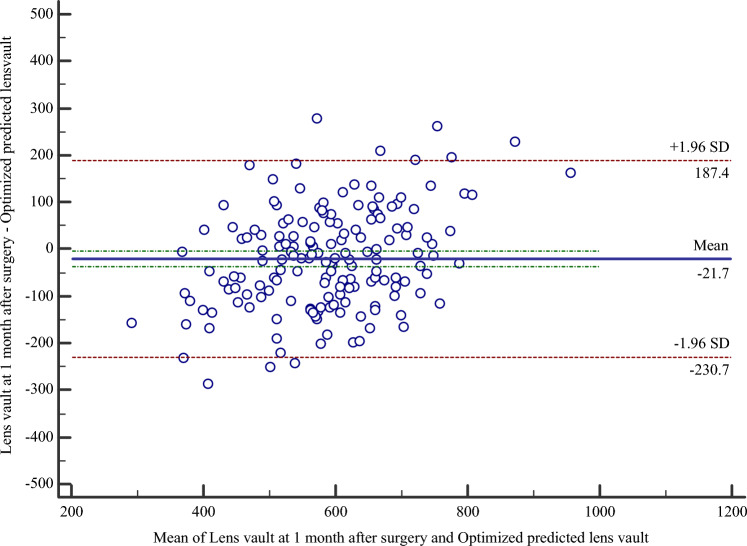
Bland–Altman plots comparing the values of achieved lens vault at one month after surgery and optimized predicted vault (The solid line in the middle represents the mean difference, and the two dotted lines above and below represent the 95% limits of agreement).

In the validation study, the mean LVp, LVop and LVa at one month after the ICL implantation were 391.94 ± 84.06 μm, 599.99 ± 101.93 μm and 579.32 ± 137.85 μm, respectively. No statistically significance was detected between LVop and LVa (*P* = 0.10). A Bland–Altman plot showed a relatively constant and systematic bias of 21.7 μm less in the lens vault at one month after surgery compared with the optimized predicted lens vault with 95% LoA of − 230.7 μm and + 187.4 μm (Fig. 2). Within the total of 170 eyes that were included in the Bland–Altman analysis, 162 eyes (95.3%) fell within the 95% LoA.

### The benefits from the optimized vault-predicting formula

**Figure 3:**
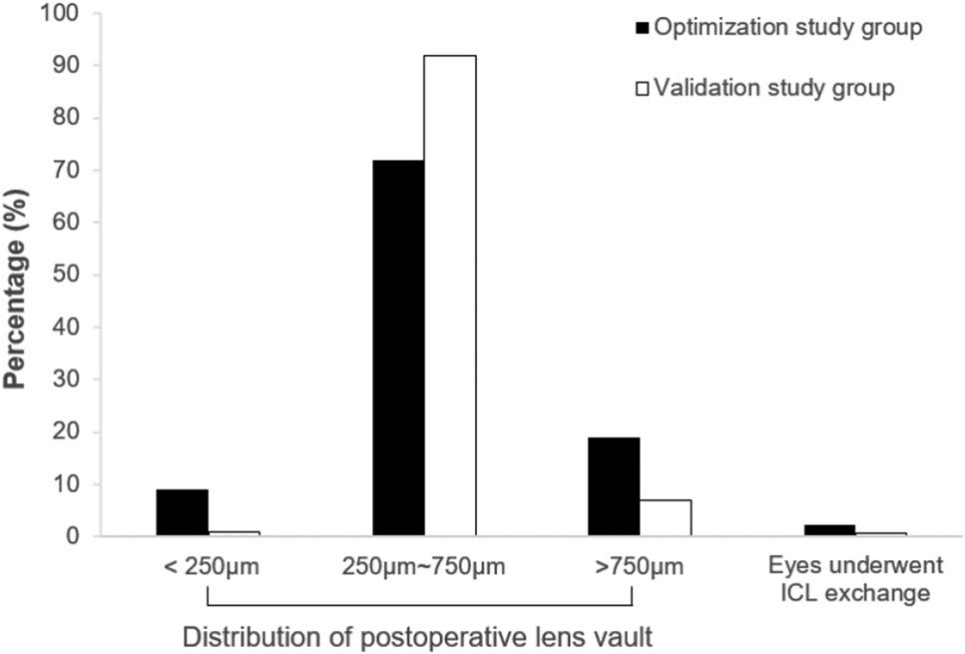
Distribution of achieved postoperative lens vault and the percentage of eyes that underwent ICL exchange due to inappropriate lens vault between the optimization and the validation study groups.

In the optimization study using OCOS, the percentages of eyes achieved low, moderate and high vaulting were 3.8%, 79.7% and 16.5%, respectively, and three eyes underwent an ICL exchanging after surgery due to an excessive lens vault (Fig. 3). In the validation study using the optimized lens vault predicting formula, 93.5% of the eyes achieved a moderate vaulting, and the rates of low vaulting and high vaulting were only 1.2% and 5.3%, respectively. Furthermore, ICL exchange was performed for just one eye (0.6%) for the unsatisfactory vaulting.

## Discussion

The Artemis Insight 100 system was introduced into our center before the beginning of the study, and the vault-predicting formula was applied according to the manufacturer’s instructions. Unfortunately, a significant difference was found between the predicted vault and the achieved vault after surgery, though we investigated that there was a statistical relationship between them. Thus the current study was conducted to optimize the vault-predicting formula, and to evaluate the accuracy of the optimized formula afterwards. Finally, the optimized vault-predicting formula was established with a good degree of fit (*R*^*2*^ = 0.68). In the validation stage, the optimized formula was proved to be effective, by which the rate of moderate post-operative vault was improved from 79.7% to 93.5%, and the rate of ICL exchange after surgery resulting from an inappropriate vault was greatly reduced. Given that a meta-analysis by Packer^21^ showed that 83.6% of eyes would have moderate vault in cases of the WTW-based ICL sizing, the results of this study are superior to the overall results from the WTW-based ICL sizing. It is worth noting that the current optimized formula is specific for measurement from Artemis Insight 100 and is not interchangeable with other diagnostics.

In the current study, 79.7% of the patients achieved moderate vault after surgery using the traditional OCOS method, which is much lower than the 93.5% achieved using the optimized vault-predicting formula. It is possible that the OCOS formula may be designed to obtain a larger postoperative vault because low post-operative low vault increases the risk of cataract in conventional ICL.

To avoid the appearance of excessively low or high vault after ICL implantation, several formulas have been created so far, but most of them only recommended the optimum ICL size. The NK formula version 1.0 developed by Nakumura et al.^24^ selected the ICL size based on the anterior chamber width (ACW) and crystal lens rise (CLR) acquired from anterior segment optical coherence tomography (AS-OCT). The fitting degree of the formula was *R*^*2*^ = 0.69 and the rate of moderate vault achieved after surgery was 71%. In the updated NK formula version 2.0^25^, the fitting degree was relatively stable, with *R*^*2*^ = 0.61, but the moderate vaulting rate was significantly improved to 91.2%. Malyugin et al.^26^ previously reported an ICL size calculation method using the iris pigment end-to-pigment end diameter, which is a component of the total angle-to-angle diameter (ATA) measurement. Even though the details of this calculation formula have not been published, the mean post-operative vault achieved using that formula was 0.53 ± 0.18 mm (range 0.24–0.84 mm), with no excessively low or high values. Dougherty et al.^19^ collected STS distance preoperatively and the vault postoperatively from 48 patients (73 eyes) of four ophthalmologists who used the VuMax-II high-frequency UBM system (Sonomed Inc., Lake Success, US). The following equation was derived from the data: ICL size = 6.624 + 0.489 (STS) + 0.264, in which the value of STS distance was the only factor determining the ICL size. The formulas mentioned above could recommend the ICL size, but were not able to predict the post-operative vault. Furthermore, there were only four ICL sizes (12.1 mm, 12.6 mm 13.2 mm, 13.7 mm) provided by the manufacture, though the recommended ICL sizes calculated by the formulas were continuous. For example, when the ICL size suggested by the formula was 12.3 mm, it was difficult to determine whether a 12.1 mm or 12.6 mm ICL ought to be implanted.

Igarashi et al.^27^ reported a vault-predicting formula, namely the KS formula, based on the measurements of ATA obtained from AS-OCT: post-operative vault (μm) = 660.9 × (ICL size [mm] -ATA [mm]) + 86.6 (adjusted *R*^*2*^ = 0.41). This formula was evaluated in a recent study^28^, and the predicted vault error (postoperative vault—predicted vault by the KS formula) was 2.6 ± 184.9 µm. Lee et al.^29^ enrolled 236 patients, which were implanted a 12.6 mm ICL. A regression formula to predict the postoperative vault was generated as: postoperative vault (mm) = -0.784 + 0.171 × ACD (mm) + 0.38 × pupil diameter (mm) + 0.017 × axial length (mm). Nevertheless, the accuracy of the formula is worth further examination because the fitting degree of the formula was relatively low (*R* = 0.144), and the location of the ICL implant was not considered. Chen et al.^30^ reported a method to predict the vault divided by different types of ICL: for V4 ICL, postoperative vault (μm) = (386.51 × ACD [mm])-781.77, and for V4c ICL, post-operative vault (μm) = (503.43 × ACD [mm])-1075.64. The adjusted *R*^*2*^ values were 0.320 and 0.297 respectively, and the number of eyes included was low (i.e., 38 eyes in V4 group and 39 eyes in V4c group). Ideally, the STS distance to which the ICL is fixed should be the most critical element to determine the post-operative lens vault. Therefore, the vault-predicting formulas based on the STS distance were introduced. In a recent study, Zhu et al.^31^ evaluated AS-OCT and UBM parameters from 83 patients (83 eyes), and investigate the relationship between them and the postoperative vault. Finally, a vault-predicting formula was generated as: postoperative vault (μm) = -1369.05 + 657.12 × ICL size (mm) − 287.41 × horizontal STS diameter (mm) − 432.50 × lens thickness (mm) − 137.33 × vertical STS diameter (mm). This formula demonstrated a good fitting degree, with *R*^*2*^ = 0.660 and adjusted* R*^*2*^ = 0.643, though the sample that was collected to develop the formula was relatively small.

The current study is the optimization of a vault-predicting formula reported by Reinstein et al.^22^, which also used the anterior segment parameters measured by the Artemis Insight 100 system, including ciliary body inner diameter (CBID), sulcus-to-sulcus lens rise (STSL), ICL size, ICL power and scotopic pupil diameter (SPD). However, the horizontal STS distance was not included in the Reinstein formula, because according to their model, STS was no longer statistically significant after inclusion of the CBID. This was supported by the finding that the lens footplates rested on or dove directly into the ciliary body in 94% of eyes compared to directly at the sulcus in only 6%^22^. In the present study, the predicted vault generated from the Reinsterin formula showed a poor relation with the achieved postoperative vault before optimization, and the result might be explained by the demographic of the population chosen in the study: the patients enrolled in the study by Reinstein et al. were Caucasian, while the participants included in the present study were Asian people. We will try to valid this optimized formula in our further studies with different human races to demonstrate its wider applicability. The optimized formula in the current study was constructed from the data of 133 eyes, which was a larger sample than the previous studies, and presented a greater fitting degree (*R*^*2*^ = 0.68) than ever before. However, we failed to perform a separate analysis for each ICL size, and we will try to accomplish it with more patients in the future.

This study has the following limitations. First, the locations of the four haptics of ICL after surgery, which could influence the lens vault, were not investigated^18^. Second, long-term changes in central vaulting because of accommodation or chronologic changes in crystalline lens thickness must also be considered^8^. Third, the accurate measurements of the anterior segment parameters obtained from Artemis Insight 100 relied on the experience of the technician. However, the examiners and surgeons in this study were set, and whether different operators or surgical habits affect the results requires further research. Finally, it costs a lot to buy Artemis Insight 100, so the device is available in few of the refractive centers all over the world.

In conclusion, a vault-predicting formula generated from the anterior segment parameters acquired by the Artemis Insight 100 VHF digital ultrasound robotic scanner was hereby optimized, and the accuracy of this optimized vault-predicting formula was also demonstrated. The optimized vault-predicting formula allows for precise vault prediction, thus enabling appropriate selection of the ICL size.

## Methods

### Design and participants

This study comprised two parts. The first part was a retrospective study to optimize the formula based on the optical parameters obtained from Artemis Insight 100 VHF digital ultrasound robotic scanner (ArcScan, Inc), by comparing the predicted to the achieved vault. The second part involved a prospective study to validate the optimized formula, in which the consistency between the post-operative vault and the vault generated from the optimized formula was analyzed. All patients who underwent ICL surgery at the Refractive Surgery Center of Henan Provincial Eye Hospital between October 2021 and May 2022 were enrolled in the vault-predicting formula optimization study. Subsequently, all patients who received ICL surgery between June 2022 and March 2023 at the same center were enrolled in the optimized vault-predicting formula validation study. All consecutive patients were included in the current study. The study protocol was approved by the Ethics Committee of Henan Provincial Eye Hospital (approval code: HNEECKY-2021-13-02). This study was conducted according to the tenets of the Declaration of Helsinki and informed consent was obtained from all participants.

The inclusion criteria were (1) 21–45 years of age, (2) ACD ≥ 2.8 mm, (3) corneal endothelial cell density (ECD) ≥ 2000/mm2, (4) clear crystal lens, and (5) the rotating angle of the ICL placement no more than 15°. The exclusion criteria were (1) other eye diseases except refractive errors, such as cataracts and glaucoma, (2) systemic diseases such as diabetes mellitus, autoimmune diseases, such as systemic lupus erythematosus and rheumatoid arthritis, or collagen diseases that may affect post-operative healing, and (3) the rotating angle of the ICL placement more than 15°.

### Preoperative measurements

Prior to surgery, all patients received a complete ophthalmologic examination, including uncorrected distance visual acuity (UDVA), manifest refraction spherical equivalent (MRSE), corrected distance visual acuity (CDVA). Anterior segment anatomy was assessed with slit-lamp microscope, IOP using a non-contact tonometer (Canon, Irvine, US), and the fundus was observed by indirect ophthalmoscopy. Moreover, the internal ACD from the corneal endothelium, the simulated keratometry, and the WTW were recorded before surgery using Pentacam HR three-dimensional panoramic analyzer (Oculus, Wetzlar, Germany). An IOL Master 700 biometer (Carl Zeiss GmbH, Oberkochen, Germany) was used to measure the axial length. SPD was measured using Scansys three-dimensional anterior segment analytic system (Mediworks, Shanghai, China). Finally, an EM-3000 corneal endothelial cell counter (Tomey Corporation, Nagoya, Japan) was used to measure corneal ECD.

### Artemis insight 100 VHF digital ultrasound scanning

**Figure 4:**
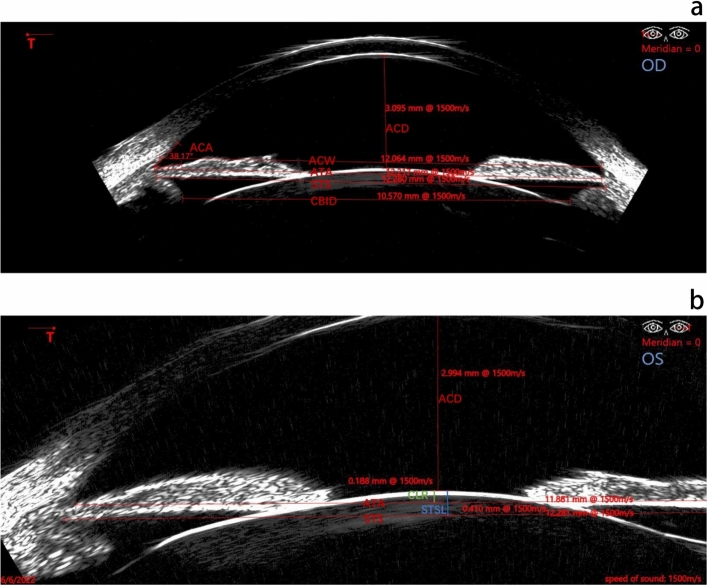
The parameters measured by Artemis Insight 100 very high-frequency digital ultrasound B scan. (**a**) Anterior chamber depth (ACD), anterior chamber angle (ACA), anterior chamber width (ACW), angle-to-angle (ATA) diameter, sulcus-to-sulcus (STS) diameter, ciliary body inner diameter (CBID); (**b**) the lens rise from the ATA plane described as crystal lens rise (CLR, shown as green) and the lens rise from the STS plane named STS lens rise (STSL, show as blue).

All participants received posterior chamber imaging and biometry using the Artemis Insight 100 VHF digital ultrasound robotic scanner by an experienced technician (DW) according to a previously described protocol^32^. Artemis scans were acquired with the patients in a sitting position with the eye coupled to the ultrasound transducer with a normal saline immersion medium contained in a disposable eye seal. During scanning, the patient gazed at a fixation light and eye position was monitored by an infrared camera. An ICL pre-operational scan set included sweeps at 0, 3, 6, 9, 351, 354, and 357 degrees, for a total of seven horizontal meridians. A minimum of two scan sets were required for each patient, providing at least 14 images to evaluate the horizontal biometry of the posterior chamber, of which the four best images were analyzed by a single observer (WH). The following parameters were measured using software calipers within the Insight 100 system to measure the following parameters: ACD, ACW defined as the sclera spur-to-spur diameter, anterior chamber angle (ACA), ATA, STS, CLR, STSL, CBID defined as the horizontal measurement from ciliary body to ciliary body (Fig. 4).

### Surgical procedure

All procedures were carried out under topical anesthesia by the same experienced surgeon (PC). Pupil dilation was initiated with compound tropicamide eye drops (Santen Pharmaceutical Co., Ltd., Osaka, Japan) instilled twice, 10 min apart. To prevent potential cyclotorsion in the supine position during Toric ICL implantation, the 0°-180° horizontal axis was preoperatively marked under the slit-lamp. Oxybuprocaine hydrochloride eye drops (Santen Pharmaceutical Co., Ltd., Osaka, Japan) were used to perform surface anesthesia. Implantation was performed through a 3.0 mm temporal incision, and the anterior chamber was filled with 15 mg/mL medical sodium hyaluronate gel (Qisheng Biological Preparation Co., Ltd., Shanghai, China). The V4c EVO ICL (STAAR Surgical AG) which comes with a central hole, was then inserted via the corneal incision using an injector cartridge (STAAR Surgical AG). After initially placing the ICL on the iris, the four haptics were tucked to the ciliary sulcus under the iris with a bespoke manipulator, and the ICL was rotated to the desired meridian 15.0° or less and its position was fixed horizontally in all cases. On positioning the ICL, the medical sodium hyaluronate gel was washed out of the anterior chamber using a balanced salt solution. Antibiotic (levofloxacin, 0.5%) and corticosteroid (loteprednol etabonate 5 mg/mL) drugs were administered topically four times daily for one week postoperatively, with gradual reduction of the dose thereafter.

### The optimization of the vault-predicting formula from artemis insight 100

The first part of the study involved examination of all patients by the Artemis Insight 100 before surgery, from which the average values of ACW, ACA, ATA, ACD, CLR, STS, STSL and CBID were measured and entered into the ICL Sizing Calculator Version 1.1 (https://www.iclsizing.com), and the predicted vault for each size (12.1 mm, 12.6 mm, 13.2 mm and 13.7 mm) were recorded. In contrast, the sizes of implanted ICL were traditionally chose by OCOS (https://ocos.staarag.ch), mainly considering the values of WTW and ACD. The patients were followed up, and the achieved lens vault at one month after the surgery was compared to the predicted vault from the corresponding ICL size. Linear regression was performed to analyze the relationship between the predicted and achieved vault, and an optimized vault-predicting formula was established.

### The validation of the optimized vault-predicting formula

The second part of the study aimed to evaluate the accuracy of the optimized vault-predicting formula according to the following protocol: the patients received the Artemis Insight 100 examination and the predicted vault for every ICL size was recorded as described in the first part. Then the predicted vault was inserted into the optimized vault-predicting formula, and the optimized predicted vault for each size, namely 12.1 mm, 12.6 mm, 13.2 mm and 13.7 mm was recorded. In contrast to the optimization study, the size of ICL implanted was selected based on the optimized vault-predicting formula, which means the size with the value of optimized predicted vault closest to 500 μm was selected to be used in the surgery. For example, if in a patient the optimized predicted vault was 488 μm for a 12.6 mm-lens and 818 μm for a 13.2 mm-lens, then the 12.6 mm-lens would have been used in the surgery. The lens vault at one month after surgery was also recorded. Finally, the degree of agreement between the achieved vault and optimized predicted vault was analyzed to validate the optimized vault-predicting formula.

### Measurement of vault

The distance between the posterior surface of the V4c ICL and anterior lens capsule (namely, the lens vault, abbreviated as LV) was measured using a swept-source OCT, CASIA SS-1000 (Tomey Corp., Nagoya, Japan). All measurements were performed by the same ophthalmologist (FQ) three times under the same indoor light for all patients, and the average values were recorded. For the LV at one month after surgery, 250–750 μm was defined as moderate vaulting, < 250 μm as low vaulting, and > 750 μm as high vaulting ^33^.

### Statistical analysis

Statistical processing of data was performed using SPSS (version 25.0) for Windows (IBM Corp., Armonk, NY, USA) and MedCalc (version 15.2.2) for Windows (MedCalc Software Ltd., Ostend, Belgium). Descriptive statistics, such as mean values and standard deviations were used to quantitatively summarize data. Continuous data conforming to the normal distribution (according to the Kolmogorov–Smirnov test) were presented as means ± standard deviations (SD). Non-normally distributed data were presented as medians (ranges). Categorical data were presented as frequencies (percentage) and analyzed using the Chi-square test or Fisher’s exact test. The relationship between the predicted vault and postoperative ICL vaulting at one month was evaluated using linear regression, and the optimized vault-predicting formula was thereafter established. Bland–Altman plots with 95% limits of agreement (LoA) were used to analyze the difference between optimized predicted vault and postoperative ICL vault at one month.

## Data Availability

The datasets generated during and/or analyzed during the current study are available from the corresponding author on reasonable request.
